# Cellular events during ovine implantation and impact for gestation

**DOI:** 10.21451/1984-3143-AR2018-0014

**Published:** 2018-08-03

**Authors:** Greg A. Johnson, Fuller W. Bazer, Robert C. Burghardt, Guoyao Wu, Heewon Seo, Avery C. Kramer, Bryan A. McLendon

**Affiliations:** 1 Department of Veterinary Integrative Biosciences, Texas A&M University, College Station, TX 77843, USA; 2 Department of Animal Science, Texas A&M University, College Station, TX 77843, USA

**Keywords:** conceptus, endometrium, placentation, pregnancy, sheep.

## Abstract

The establishment of pregnancy in sheep includes elongation of the blastocyst into a filamentous conceptus, pregnancy recognition, production of histotroph, attachment of the conceptus to the endometrium for implantation, and development of synepitheliochorial placentation. These processes are complex, and this review describes some of the molecular events that underlie and support successful pregnancy. The free-floating sheep blastocyst elongates into a filamentous conceptus and metabolizes, or is responsive to, molecules supplied by the endometrium as histotroph. Amongst these molecules are SPP1, glucose and fructose, and arginine that stimulate the MTOR nutrient sensing system. The placental trophectoderm of elongating conceptuses initiate pregnancy recognition and implantation. The mononucleate cells of the trophectoderm secrete IFNT, which acts on the endometrial LE to block increases in estrogen receptor α to preclude oxytocin receptor expression, thereby preventing oxytocin from inducing luteolytic pulses of PGF2α. In addition, IFNT increases expression of IFN stimulated genes in the endometrial stroma, including ISG15, a functional ubiquitin homologue. Implantation is the initial step in placentation, and includes sequential pre-contact, apposition, and adhesion phases. Implantation in sheep includes downregulation of Muc1 and interaction of GLYCAM1, galectin 15 (LGALS15) and SPP1 with lectins and integrins (αvβ3). Sheep have synepitheliochorial placentation in which mononucleate trophectoderm cells fuse to form binucleate cells (BNCs). BNCs migrate and fuse with endometrial LE cells to form trinucleate syncytial cells, and these syncytia enlarge through continued BNC fusion to form syncytial plaques that form the interface between endometrial and placental tissues within the placentome. The placentae of sheep organize into placentomal and interplacentomal regions. In placentomes there is extensive interdigitation of endometrial and placental tissues to provide hemotrophic nutrition to the fetus. In interplacentomal regions there is epitheliochorial attachment of endometrial LE to trophectoderm, mediated through focal adhesion assembly, and areolae that take up histotroph secreted by endometrial GE.

## Introduction

Domestic animal models for research are generally underappreciated (Roberts *et al*., 2009); however, sheep offer unique characteristics of pregnancy, as compared to rodent or primate models, and studies of sheep have provided significant insights into the physiology of implantation including: 1) elongation of the blastocyst into a filamentous conceptus; 2) the protracted peri-implantation period of pregnancy when the conceptus is free within the uterine lumen requiring extensive paracrine signaling between conceptus and endometrium, as well as nutritional support provided by uterine secretions; 3) a protracted and incremental attachment cascade of trophectoderm to endometrial epithelium during implantation; and 4) development of a synepitheliochorial placenta that utilizes extensive endometrial and placental vasculatures for hemotrophic nutrition, and placental areolae for histotrophic support of the developing fetuses. Our understanding of the complex molecular events that underlie successful pregnancy recognition in ruminants, the attachment phase of implantation that occurs across all species, and placentation in livestock species have been, and will likely continue to be, advanced by studies of sheep as agricultural and biomedical research models.

## Elongation of the blastocyst into a filamentous conceptus

**Figure 1:**
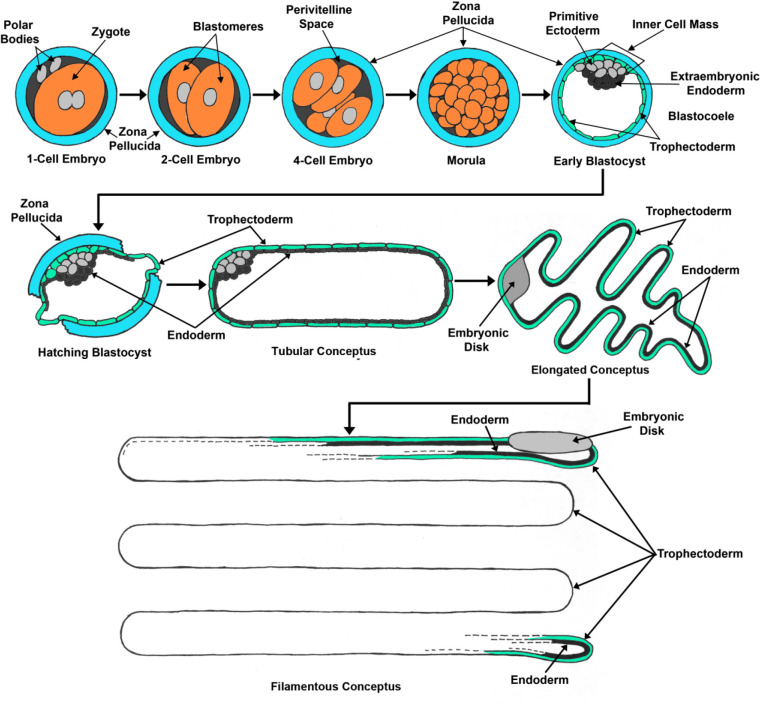
Elongation of the blastocyst into a filamentous conceptus. Sheep embryos enter the uterus at about day 3 or 4, develop into spherical blastocysts and then, after hatching from the zona pellucida, transform from spherical to tubular and filamentous conceptuses (embryos and associated placental membranes) between days 12 and 15 of pregnancy, with placental membranes extending into the contralateral uterine horn between days 16 and 20 (Bazer *et al*., 2005). Conceptus elongation ensures maximum area of surface contact between the conceptus trophectoderm and endometrial luminal epithelium (LE).

The early stages of embryonic development in sheep proceed in a manner similar to other mammalian species (Fig. 1). After fertilization within the oviduct, the zygote undergoes the first cleavage division to form the 2-cell embryo, and cleavage divisions continue through the 8-16 cell stage, when transcriptome activation occurs. These divisions culminate in formation of the solid mass of cells, called the morula (16-32 cells), that remains encased in the zona pellucida of the original oocyte. The morula remains in the oviduct before entering the uterus on day 3 or 4 in sheep. Embryos of most species fail to develop beyond the early blastocyst stage if confined to the oviduct, and it is speculated that is due to the absence of critical factors required for embryonic development that are supplied by the uterus. The developing embryo next forms the blastocyst by day 6. At this point the pluripotent blastomeres begin to differentiate into the inner cell mass (ICM) and trophectoderm to begin the cell lineages that will eventually become the embryo/fetus proper (primitive ectoderm, mesoderm and endoderm), and the placental trophectoderm/chorion, respectively, and are collectively termed the conceptus (Bazer *et al*., 2005). The blastocyst hatches out of the zona pellucida between days 8 and 9 (200 µm in diameter and containing about 300 cells), and increases in size (400- 900 µm in diameter and containing about 400-900 cells) before undergoing a rapid morphological transition called elongation (Fig. 1). The small spherical conceptus grows into a tubular form by day 11, followed by a phase of rapid growth and elongation between days 12 and 16 to form the mature filamentous conceptus (10-22 mm on day 12, 10 cm on day 14, and 25 cm on day 17). During the early elongation period, the conceptus remains unattached to the uterine endometrium and dependent on nutrients in the uterine lumen. Conceptus elongation substantially increases the surface area of placental trophectoderm that will subsequently directly attach to, and interact in a close paracrine manner with the uterus. This provides increased surface area for nutrient exchange between the conceptus and endometrium, and maximize the paracrine effects of the conceptus to prevent luteolysis for pregnancy recognition (Spencer *et al*., 2004a).

As sheep conceptuses elongate they metabolize, or are responsive to, significant concentrations of molecules supplied in the form of histotroph, a complex mixture of hormones, enzymes, growth factors, cytokines, transport proteins, adhesion factors, nutrients and other substances that plays roles in conceptus nourishment, implantation and placentation. Mammalian cell growth in general, and particularly in cells of the conceptus, is regulated by growth factors and the availability of nutrients. The mechanistic mammalian target of rapamycin (MTOR) cell signaling pathway plays an important role in regulation of cell growth and metabolism in response to growth factors and nutritional status to affect biological and physiological responses of cells and organs. The MTOR pathway is a nutrient sensing system stimulated by molecules that include Secreted Phosphoprotein 1 (SPP1), also called osteopontin (OPN), the hexose sugars glucose and fructose, and select amino acids, including arginine (Nielsen *et al*., 1995; Martin and Sutherland, 2001; Kim *et al*., 2010), to support blastocyst/conceptus development. The MTOR cell signaling pathway is a prominent component of the peri- implantation intra-uterine environment in sheep (summarized in Bazer *et al*., 2012b). FK506-binding protein 12-rapamycin complex-associated protein 1 (FRAP1), G protein β subunit-like (LST8), mitogen- activated protein kinase-associated protein 1 (MAPKAP1), regulatory-associated protein of mechanistic mammalian target of rapamycin (RAPTOR), rapamycin-insensitive companion of mechanistic mammalian target of rapamycin (RICTOR), tuberous sclerosis 1 (TSC1), tuberous sclerosis 2 (TSC2), ras homolog enriched in brain (RHEB) and eukaryotic translation initiation factor 4E-binding protein 1 (EIF4EBP1) are expressed by the endometrium and trophectoderm of sheep conceptuses between days 13 and 18 of pregnancy, and increases in abundance of RICTOR, RHEB, EIF4EBP1 and RHEB are coordinate with rapid growth and development of ovine conceptuses during the peri-implantation period (Gao *et al*., 2009a). Further, progesterone (the hormone of pregnancy) and interferon tau (IFNT; the pregnancy recognition signal in sheep) stimulate expression of RHEB and EIF4EBP1 in the endometrium of sheep (Gao *et al*., 2009a), and MTORC1 is abundant in the cytoplasm, and phosphorylated MTOR is abundant in the nuclei of sheep trophectoderm cells (oTr; Kim *et al*., 2011b).

SPP1 is a prominent component of the uterine environment during pregnancy in sheep (Johnson *et al*., 2014). SPP1 induces motility in human trophectoderm cells through MTOR signaling (Al-Shami *et al*., 2005) and rapamycin inhibits F-actin reorganization and phosphorylation of focal adhesion proteins stimulated by IGF1 (insulin-like growth factor 1) such as focal adhesion kinase (FAK; Liu *et al*., 2008). Those results suggested a role for SPP1-induced MTOR complex signaling during conceptus elongation in sheep. Therefore, we identified relationships and crosstalk between multiple membrane and intracellular cell signaling cascades activated by SPP1, including MTOR and integrin binding to ovine trophectoderm cells. These interaction potentially participate in controlling proliferation, migration and attachment of oTr cells of the conceptus to endometrial luminal epithelium (LE) during the peri-implantation period of pregnancy (Kim *et al*., 2010). Results of these studies demonstrated that SPP1 binds αvβ3 and possibly α5β1 integrin heterodimers to induce focal adhesion assembly, a prerequisite for adhesion and migration of oTr cells, through activation of: 1) P70S6K via crosstalk between FRAP1/MTOR and MAPK pathways; 2) MTOR, phosphoinositide 3 kinase (PI3K), MAPK3/MAPK1 (ERK1/2) and MAPK14 (P38) signaling to stimulate oTr cell migration; and 3) focal adhesion assembly and myosin II motor activity to induce migration of oTr cells (Kim *et al*., 2010).

Arginine is a prominent component of the uterine environment during pregnancy in sheep, and is highly stimulatory to proliferation, migration and protein synthesis in an established oTr ovine cell line (Kim *et al*., 2011a, b); therefore, pathways whereby arginine mediates it effects in oTr cells were studied. The major findings were that arginine: 1) increases phosphorylation of ribosomal protein S6 kinase (RPS6K) in a dose dependent manner; 2) increases phosphorylated forms of RAC-alpha serine/threonine- protein kinase (AKT1), RPS6K and RPS6 over basal levels 3) increases nuclear phosphorylated RPS6K and cytoplasmic phosphorylated RPS6; and 4) stimulates proliferation and migration of oTr cells (Kim *et al*., 2011a). Further, phosphorylation of RPS6K and RPS6 is blocked by inhibitors of both PI3K and MTOR cell signaling, and L-arginine, but not D-arginine, activates MTOR cell signaling via phosphorylation of RPS6K and RPS6 (Kim *et al*., 2011b). Experiments were also conducted to determine whether effects of arginine on cell proliferation are due to its metabolism to nitric oxide (NO) via NO synthase 1/2 (NOS1/NOS2) or due to its metabolism by arginase to ornithine which is converted by ornithine decarboxlylase 1 (ODC1) to the polyamines putrescine, spermidine and spermine (Kim *et al*., 2011c). Two NO donors, S-nitroso-N-acetyl-DL- penicillamine (SNAP) and diethylenetriamine NONOate (DETA), increased proliferation of oTr cells as did putrescine. Both N-nitro-L-arginine methyl ester hydrochloride (L-NAME: a NOS inhibitor to reduce NO synthesis) and N-hydroxy-nor-L-arginine (nor-NOHA; an arginase inhibitor to block synthesis of putrescine) decreased oTr cell proliferation. Therefore, both NO and polyamines can stimulate proliferation and migration of trophectoderm cells essential to conceptus elongation in sheep. In practice, exogenous administration of arginine enhances embryonic survival in sheep, as in other mammals such as pigs, mice and rats (Wu *et al*., 2013; Wu *et al*., 2017).

In sheep uteri, total recoverable glucose increases 12-fold between days 10 and 15 of pregnancy, glucose transporter 1 (GLUT1) increases in the endometrium of ovariectomized sheep in response to progesterone and an additional 2.1-fold in response to IFNT, and GLUT3 is expressed by conceptus trophectoderm (Gao *et al*., 2009b). In addition, fructose is detected in uterine flushings of pregnant gilts as early as day 12 of pregnancy and maximum concentrations of fructose rise to between 11.1 and 33 mM (Bazer *et al*., 2011; Kim *et al*., 2012). Further, the glucose that is not metabolized via metabolic pathways for production of ATP (glycolysis) or pentose phosphate pathway products in sheep is converted to fructose by the trophectoderm. Using our oTr cells we found: 1) that fructose and glucose are equivalent in stimulating cell proliferation via the MTOR pathway; 2) that phosphorylation of RPS6K and EIF4EBP1 in response to fructose requires both PI3K and MTOR, and glutamine-fructose-6-phosphate transaminase 1 (GFPT1); and 3) that inhibition of the hexosamine biosynthesis pathway by azaserine blocks MTOR- RPS6K and MTOR-EIF4EBP1 signaling and the ability of fructose to stimulate proliferation of oTr cells (Wang *et al*., 2016a). We now propose that fructose and glucose support rapid growth and development of the sheep placenta through a process whereby trophectoderm cells enter into the serinogenesis pathway for one-carbon metabolism for synthesis of: 1) purines, required for the synthesis of nucleotides and nucleic acids; 2) thymidine, required for the synthesis of DNA; and 3) S-adenosylmethionine (SAM), the principal biological methylating agent for epigenetic modifications. Due to the possibility that oTr cells in culture may have an altered phenotype, effects of glucose were evaluated using day 16 sheep conceptus explant cultures. Glucose stimulated increased abundance of total and phosphorylated forms of the MTOR cell signaling pathway proteins, as well as ODC1, NOS2 and GTP cyclohydrolase 1 (GCH1) proteins (Kim *et al*., 2011c).

## Pregnancy recognition, production of histotroph, and induction of classical IFN stimulated genes (ISGs) in the endometrium

**Figure 2:**
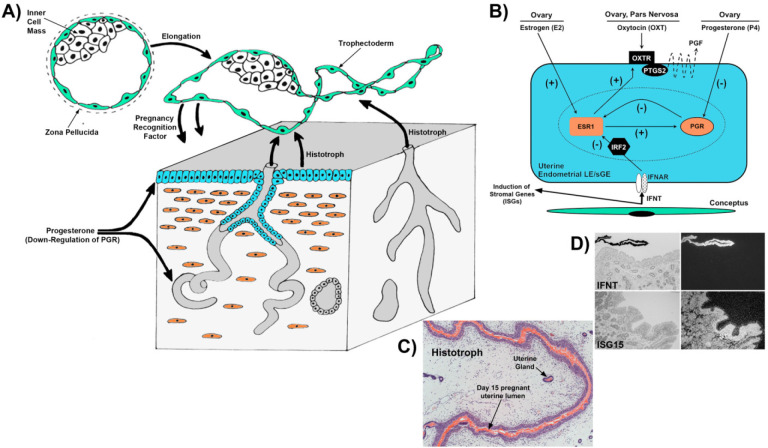
Pregnancy recognition, production of histotroph, and induction of classical IFN-stimulated genes. A) Generalized diagram of paracrine effects between the conceptus (primarily the pregnancy recognition signal IFNT) and the endometrium. B) Model for pregnancy recognition in the sheep in which IFNT silences expression of estrogen receptor α and this prevents expression of estrogen dependent expression of oxytocin receptor. C) Hematoxylin and Eosin (H&E) staining illustrating protein (histotroph) in the endometrial lumen of a pregnant sheep. D) *In situ* hybridization illustrating the expression of IFNT by conceptus trophectoderm and induction of ISG15 in the endometrium.

The peri-implantation period of mammals is complex, involving the overlapping events of pregnancy recognition and remodeling for implantation/placentation necessary for embryonic survival during early pregnancy. During early pregnancy in sheep, the mononuclear cells of the placental trophectoderm synthesize and secrete IFNT, the signal for maternal recognition of pregnancy (Spencer *et al*., 2004a). IFNT acts on the endometrial LE and superficial glandular epithelium (sGE) to block increases in transcription of estrogen receptor α to preclude estrogen receptor α interactions with Sp1 and/or AP-1 that otherwise stimulate oxytocin receptor expression, thereby preventing oxytocin from inducing release of luteolytic pulses of prostaglandin F_2_α (Fig. 2A and 2B; Fleming *et al*., 2005). This results in maintenance of the corpus luteum, the source of progesterone required for successful pregnancy (Spencer *et al*., 2004a). During the period of pregnancy recognition, progesterone down-regulates expression of progesterone receptors in the endometrial LE and glandular epithelium (GE). The loss of progesterone receptor expression by these epithelia appears to be prerequisite for progesterone to stimulate production and secretion of histotroph, a mixture of hormones, growth factors, nutrients, and other substances required for growth and development of the conceptus and implantation (Fig. 2A and 2C; Bazer *et al*., 2012a). The consensus is that the role of progesterone in producing histotroph is mediated via progesterone receptor (PGR); however, PGR are not expressed in uterine epithelia that secrete histotroph during the peri-implantation period (Spencer *et al*., 2007). It is clear that regulation of gene expression in the endometrium by progesterone during the peri-implantation period is complex. Induction of genes in uterine epithelia may require that progesterone down-regulate PGR, thereby eliminating PGR- dependent inhibition of expression of progesterone- regulated genes. However, another explanation is that progesterone induction of expression of genes in uterine epithelia is mediated by a paracrine-acting factor(s) (progestamedin) produced by the PGR-positive stromal cells (Spencer *et al*., 2007). The endometrial GE have primary responsibility for the production of histotroph in sheep. Indeed, uterine gland knockout UGKO) ewes lack endometrial GE and exhibit a peri-implantation defect and loss of pregnancy that is associated with the absence of the synthesis and secretion of key components of histotroph (Gray *et al*., 2001, 2002).

In addition to its antiluteolytic effects, IFNT also increases expression of several ISGs in the stroma and GE of the sheep endometrium (Fig. 2A and 2D). The list of ISGs known to be upregulated in the endometrium of sheep has grown from one (ISG15; Johnson *et al*., 1999c), to 15 that have actually been localized to the endometrial stroma of sheep (reviewed in Spencer *et al*., 2007; genes listed in Johnson *et al*., 2008; Hansen *et al*., 2017). Although the temporal and spatial expression within the endometrial stroma of pregnant sheep varies slightly among genes, they for the most part follow the expression pattern first described for ISG15 (Johnson *et al*., 1999c). ISG15 is first detectable in the endometria LE and stratum compactum stroma on day 13 of pregnancy (immediately prior to implantation); then expression extends to the stratum spongiosum stroma by day 15 (time of implantation). Expression is maintained throughout the stroma through day 25, then declines by day 30 of pregnancy, with expression limited to patches of the stratum compactum stroma along the maternal-conceptus interface where it remains throughout pregnancy (Johnson *et al*., 1999c; Joyce *et al*., 2005). Interestingly, most classical ISGs, including ISG15, are not induced or upregulated by IFNT in the endometrial LE of the sheep endometrium during early pregnancy, most likely due to the expression of interferon regulatory factor 2 (IRF2) in the endometrial LE (Choi *et al*., 2001). IRF2, a potent transcriptional repressor of ISGs (Taniguchi *et al*., 2001), is expressed specifically in the endometrial LE and represses activity of IFN-stimulated response element (ISRE)-containing promoters. It is hypothesized that the lack of ISG induction and silencing of ISGs, such as major histocompatability complex class 1 (MHC1) and beta 2 microglobulin (B2M), may be involved in the prevention of immune rejection of the semi-allogeneic conceptus (Choi *et al.* 2003; Joyce *et al*., 2008). MHC1 molecules are polymorphic cell surface glycoproteins expressed on most somatic cells that present peptide antigens derived from self proteins or from proteins of intracellular pathogens to cytotoxic T lymphocytes; therefore, they are involved in immune recognition of foreign pathogens and transplanted allogeneic tissues. The laws of transplantation biology dictate rejection of the conceptus as a semiallogeneic tissue with paternal as well as maternal histocompatibility antigens. It is reasonable to project that downregulation of these molecules in the endometrium benefits pregnancy by blinding cytotoxic T lymphocytes to the presence of the foreign alloantigens within the conceptus trophectoderm to prevent immune rejection (Joyce *et al*., 2008).

At present, we can only speculate on the roles of classical ISGs within the pregnant endometrium. The best characterized of these genes, ISG15 is a functional ubiquitin homologue that has the C-terminus Leu-Arg- Gly-Gly amino acid sequence common to ubiquitin, allowing conjugation to intracellular proteins (Haas *et al*., 1987). Conjugation of proteins either targets them for rapid degradation in the proteasome, or stabilizes the proteins for long-term modification (Wilkinson, 2000). ISG15 does indeed form stable conjugates with endometrial proteins of sheep and cows, indicating a biologically active molecule that is responsive to the IFNT signal from the trophectoderm that can temporally target proteins for pregnancy-associated regulation and/or modification (Johnson *et al*., 1998; Joyce *et al*., 2005). ISG15 is expressed in the decidua of mice (Austin *et al*., 2004; Bebington *et al*., 1999a), and women (Bebington *et al*., 1999b), and in the endometrial stroma of pigs (Johnson *et al*., 2009). The decidua has been hypothesized to play roles in hormone secretion, conceptus nutrition, fetal allograft protection, uterine remodeling, limiting of conceptus trophectoderm invasion, and as a defender against infectious inflammatory insults through generation of a local cytokine environment. Therefore it is possible that ISG15 conjugation/ISGylation in decidua is involved in one or more of these processes (Hansen and Pru, 2014).

## Attachment cascade of the conceptus to the endometrium for implantation

**Figure 3:**
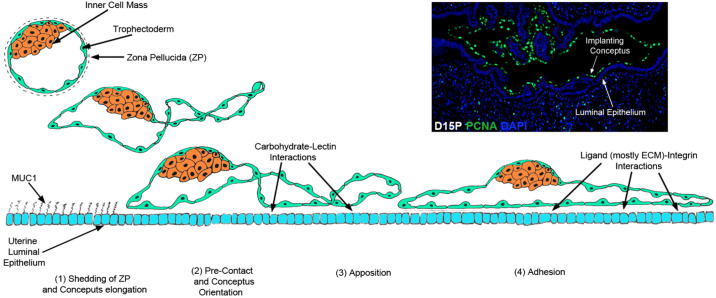
Attachment cascade of the conceptus to the endometrium for implantation. A generalized diagram of implantation in epitheliochorial and synepitheliochorial species. Implantation in sheep extends from days 11-16 and includes four phases that overlap and involve increasingly complex interactions between conceptus trophectoderm and endometrial luminal epithelium (LE). The inserted picture depicts immunostaining of proliferating trophectoderm cells (green color) at an implantation site. The current consensus for the attachment cascade in sheep includes downregulation of mucin 1 (MUC1) across the entire endometrial surface, which unmasks glycosylation dependent cell adhesion molecule 1 (GLYCAM1), galectin-15 and SPP1 for interaction with lectins and integrins. Initial attachment is likely mediated by GLYCAM1 and LGALS15, and firm attachment is likely mediated by SPP1 and the αvβ3 integrin receptor (Spencer *et al*., 2004).

Implantation is the initial step in placentation, and proper attachment of the conceptus to the endometrium is critical to successful pregnancy (Denker, 1993; Burghardt *et al*., 2002). The process of implantation is highly synchronized, requiring reciprocal secretory and physical interactions between a developmentally competent conceptus and the endometrium during a restricted period of the uterine cycle termed the window of receptivity. These initial interactions between the apical surfaces of endometrial LE and conceptus trophectoderm cells follow an attachment cascade. This attachment cascade is similar to that observed during the extravasation of white blood cells from the vasculature through the endothelium and into connective tissues, and includes sequential non- adhesive or pre-contact, apposition, and adhesion phases, resulting in formation of a placenta that supports fetal-placental development throughout pregnancy (Fig. 3; Carson *et al*., 2000). Conceptus attachment first requires the removal of large mucins from the glycocalyx of the endometrial LE that would otherwise sterically inhibit adhesion (Aplin *et al*., 2001). The removal of these mucins allows for direct physical interactions between carbohydrates and lectins at the apical surfaces of the opposing endometrial LE and conceptus trophectoderm cells (Kimber *et al*., 1995). These low affinity contacts are then replaced by firm adhesion between integrins and extracellular matrix (ECM) proteins (Burghardt *et al*., 2002; Lessey, 2002).

The term implantation is somewhat a misnomer for the sheep, but nevertheless, it is used to describe the initial stages of placentation in the livestock species. In sheep, the filamentous conceptus is closely associated with the endometrial LE and appears to be immobilized within the uterine lumen by day 14, although the conceptus can still be recovered intact from the uterus by lavage with only superficial damage. Apposition begins near the inner cell mass, and spreads towards the ends of the elongated conceptus, and by day 16 the conceptus trophectoderm is firmly attached to the endometrial LE with significant interdigitation between the microvilli on endometrial LE and conceptus trophectoderm cells, as well as between placental papillae that extend down into the lumen of the ducts of endometrial glands. The conceptus attaches to both the caruncular and intercaruncular regions of the endometrium, and attachment is complete by day 22 (Guillomot *et al*., 1981; Spencer *et al*., 2004b). The current consensus for the attachment cascade in sheep includes downregulation of mucin 1 (MUC1) across the entire endometrial surface, which unmasks glycosylation dependent cell adhesion molecule 1 (GlyCAM-1), LGALS15 and SPP1 for interaction with lectins and integrins. Initial attachment is likely mediated by GLYCAM1 and galectin-15, and firm attachment is likely mediated by SPP1 (Spencer *et al*., 1999, 2004b; Johnson *et al*., 1999a, 2001, 2003, 2014; Gray *et al*., 2004; Muñiz *et al*., 2006). Although progesterone downregulates Muc1 in pigs, progesterone does not appear to decrease Muc1 expression on the apical surface of the endometrial LE of sheep (Bowen *et al*., 1996; Johnson *et al*., 2001). Integrins are constitutively present on endometrial LE and conceptus trophectoderm during the peri-implantation period (Johnson *et al*., 2001). LGALS15 expression is induced by progesterone and LGALS15 expression is further increased by IFNT (Gray *et al*., 2004). SPP1 expression is induced by progesterone (Johnson *et al*., 2000).

Interestingly, SPP1 is not directly synthesized by the endometrial LE of sheep, but is a component of histotroph secreted from the endometrial GE into the uterine lumen of pregnant ewes as early as day13. It is not secreted by the endometrial GE of cyclic ewes (Johnson *et al*., 1999b, 1999a). SPP1 mRNA is expressed by some endometrial GE as early as day 13, and is present in the majority of the endometrial GE by day 19 of gestation (Johnson *et al*., 1999b). Progesterone induces expression of SPP1 in the endometrial GE, and induction is associated with a loss of progesterone receptor in the endometrial GE (Johnson *et al*., 2000). Analysis of uterine flushings from pregnant sheep identified a 45 kDa fragment of SPP1 with greater binding affinity for αvβ3 integrin receptor than native 70 kDa (Johnson *et al*., 2000; Senger and Perruzzi, 1996). This 45-kDa SPP1 cleavage fragment is exclusively, continuously, and abundantly present along the apical surface of the endometiral LE, on the apical surface to the trophectoderm/chorion, and along the entire uterine-placental interface through day 120 of pregnancy (Johnson *et al*., 2003). Comparison of the spatial distribution of SPP1 mRNA and protein by *in situ* hybridization and immunofluorescence analyses of cyclic and pregnant sheep uterine sections has provided significant insight into the physiology of endometrial SPP1 during pregnancy. SPP1 mRNA increases in the endometrial GE during the peri-implantation period; however, it is not present in endometrial LE or conceptus trophectoderm (Johnson *et al*., 1999b). In contrast, immunoreactive SPP1 protein is present at the apical surfaces of endometrial LE and GE, and on conceptus trophectoderm where the integrin subunits αv, α4, α5, β1, β3, and β5 could contribute to the assembly of several SPP1 receptors including αvβ3, αvβ1, αvβ5, α4β1, and α5β1 heterodimers which are expressed constitutively on the apical surfaces of conceptus trophectoderm and endometrial LE (Johnson *et al*., 1999a, 2001). Affinity chromatography and immunoprecipitation experiments have determined whether αv, α4, α5, β1, β3, β5, and β6 integrins expressed by oTr cells directly bind SPP1. Successful immunoprecipitation of labeled oTr integrins occurred with antibodies to αv and β3 integrin subunits, as well as an antibody to the integrin αvβ3 heterodimer. Antibody to the αv integrin subunit also precipitated a β chain, presumed to be the β3 integrin subunit, as an antibody to the β3 integrin subunit precipitated an α chain at the same relative size as the bands precipitated by an antibody to the αvβ3 heterodimer. Thus, the αvβ3 integrin on oTr cells binds SPP1 (Kim *et al*., 2010). SPP1 binding to the αvβ3 integrin receptor induced *in vitro* focal adhesion assembly, a prerequisite for adhesion and migration of trophectoderm, through activation of: 1) P70S6K via crosstalk between FRAP1/MTOR and MAPK pathways; 2) MTOR, PI3K, MAPK3/MAPK1 (Erk1/2) and MAPK14 (p38) signaling to stimulate trophectoderm cell migration; and 3) focal adhesion assembly and myosin II motor activity to induce migration of conceptus trophectoderm cells (Kim *et al*., 2010). Recently we reported that SPP1 binds integrins to form focal adhesions that activate the MTORC2 pathway for cytoskeletal reorganization in both adhered and migrating oTr cells, and that SPP1 cooperates with arginine to increase oTr cell adhesion and migration (Wang *et al*., 2016b). Collectively, results indicate that SPP1 binds αvβ3 integrin receptor to activate cell signaling pathways that act in concert to mediate adhesion, migration and cytoskeletal remodeling of conceptus trophectoderm cells essential for expansion and elongation of conceptuses and their attachment to endometrial LE for implantation (Johnson *et al*., 2014).

## Development of synepitheliochorial placentation

**Figure 4:**
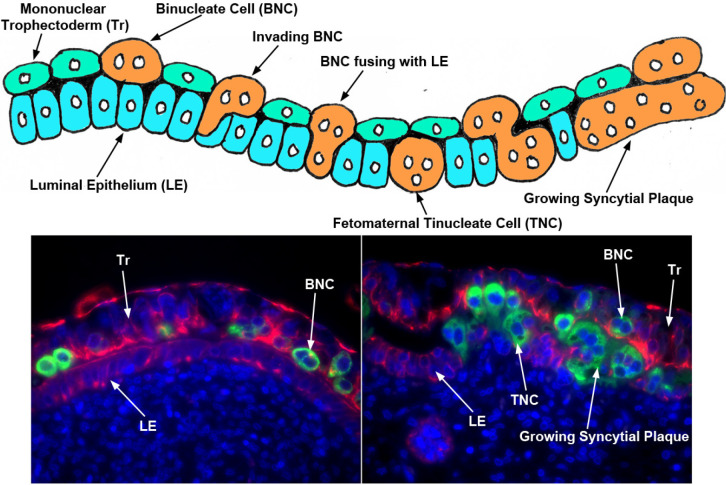
Syncytialization at the uterine-placental interface of sheep (Wooding *et al*., 1984; Wooding and Burton, 2008). Illustrated are a cartoon of the current consensus for syncytia formation in the sheep and immunofluorescence staining for pregnancy associated glycoprotein (PAG; Szafranska *et al*., 1995); stains BNCs, TNCs and syncytial plaques green) and CDH1 (epithelial cadherin (E-Cad); stains mononuclear Tr and endometrial LE cells red).

As ruminants, sheep demonstrate synepitheliochorial placentation in which fusion of conceptus trophectoderm with endometrial LE occurs. Two morphologically and functionally distinct cell types, mononucleate trophectoderm cells and binucleate trophoblast giant cells (BNCs), are present in the trophectoderm of ruminant placentae (Fig. 4). The mononucleate cells constitute the majority of the trophectoderm cells and BNCs begin to differentiate from the mononucleate trophectoderm cells in concert with trophectoderm outgrowth during conceptus elongation. BNCs first appear between days 14 and 16 of gestation in sheep conceptuses, and comprise 15-20% of the trophectoderm during the apposition and attachment phases of implantation. BNCs migrate and fuse with individual endometrial LE cells to form trinucleate syncytial cells, beginning on about day 16 of pregnancy in sheep, thereby assimilating the endometrial LE. The syncytia of sheep subsequently enlarge through continued BNC migration and fusion to form syncytial plaques (Fig. 4). The syncytial plaques are conceptus-maternal hybrid cells that are composed of endometrial LE cells and conceptus BNCs, and they eventually form the epithelial interface between endometrial and placental tissues within the placentome. In sheep, the syncytial plaques are a consistent feature in the placentomes throughout pregnancy (Wooding, 1984; Wooding and Burton, 2008).

**Figure 5:**
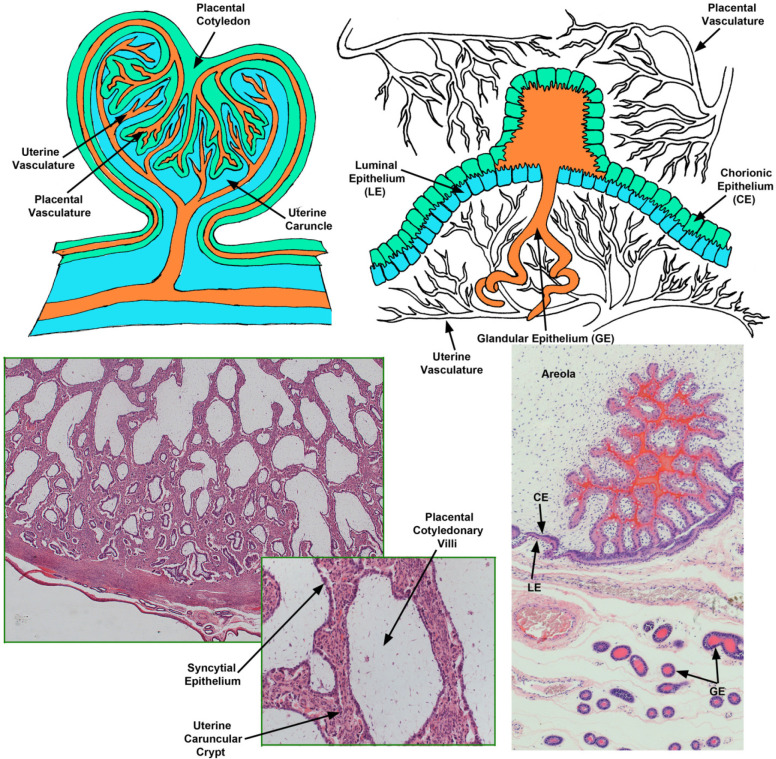
The endometrial-placental interface of mature placentation in the sheep illustrating the placentome and the areola. Illustrated are cartoons depicting the structure of the sheep placentome (Top Left) and areola (Top Right), and H&E staining of a paraffin embedded thin section of a placentome (Bottom Left) and an areola (Bottom Right).

Following successful elongation of the conceptus, trophectoderm outgrowth, and implantation, the placentae of sheep organize into placentomal and interplacentomal regions (Fig. 5). During placentome development, highly branched villous placental folds, termed cotyledons, initially form by day 30 of gestation in sheep. Cotyledonary chorioallantoic villi lined by syncytial plaques then begin to protrude into crypts in the maternal endometrial caruncular tissue (aglandular areas of endometrium consisting of stroma covered by a single layer of epithelium), resulting in extensive interdigitation of endometrial and placental tissues by day 40. Placentomes provide a conduit for hemotrophic nutrition to the fetus where maternal and placental blood vessels are in close proximity for exchanging oxygen and micronutrients, and there is a close correlation between the placentomal mass and the birthweight of the fetus. In contrast, interplacentomal areas exhibit epitheliochorial attachment of endometrial LE to conceptus trophectoderm, and contain areolae that take up histotroph secreted by the endometrial GE for transport into placental vasculature that rings each of the areolae (Fig. 5; Wooding and Burton, 2008).

**Figure 6:**
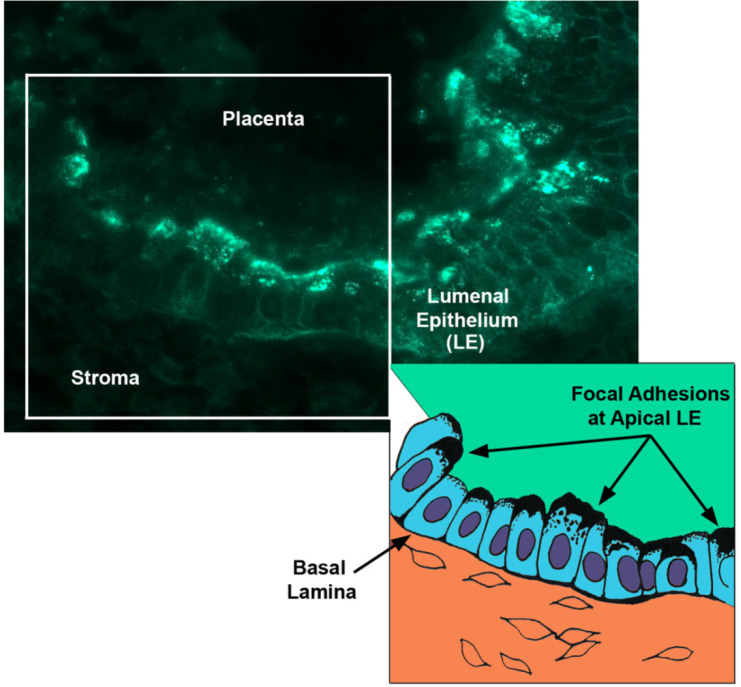
Focal Adhesions at the endometrial-placental interface of interplacentomal regions of placentation in the sheep (Burghardt *et al*., 2009). Illustrated is immunofluorescence staining (Top Panel) for αv integrin and a cartoon (Bottom Panel) depicting the localization of the focal adhesions that form at the interface between endometrial LE cells and the chorion.

Focal adhesions (FAs), the hallmark of activated integrins, are prominent structures of cells grown in culture; however, they are rarely observed *in vivo*. It is noteworthy that large aggregations of FA- associated proteins, that have been interpreted to be *in vivo* FAs, are present at the endometrial-placental interface of sheep (Johnson *et al*., 2003; Burghardt *et al*., 2009; Fig. 6). By day 40 of pregnancy in sheep, the punctate apical surface staining of integrin receptor subunits identified in peri-implantation endometrial LE and conceptus trophectoderm is replaced by scattered large aggregates of αv, α4, β1, and β5 subunits in interplacentomal endometrial LE and conceptus trophectoderm/chorion cells (Johnson *et al*., 2001; Burghardt *et al*., 2009). Integrin aggregates are observed only in the gravid uterine horns of unilaterally pregnant sheep, demonstrating a requirement for conceptus trophectoderm/chorion attachment to endometrial LE, and aggregates increase in number and size through day 120 of pregnancy. In some regions of the interplacentomal interface, greater subunit aggregation occurs on the endometrial side, in other regions it is predominant on the placental side; whereas in some other regions, both endometrial and placental epithelia exhibit prominent FAs. However, by day 120 of pregnancy, extensive FAs are present along most of the endometiral- placental interface (Burghardt *et al*., 2009). The placentomes, which provide hemotrophic support to the fetus and placenta, exhibited diffuse immunoreactivity for these integrins compared with interplacentomal regions possibly due to extensive folding at this interplacentomal interface and the 3D nature of the ECM within placentomes. It is noteworthy that interplacentomal endometrial stroma, only within the gravid horn of unilaterally pregnant sheep, also exhibits robust but punctate immunostaining for αv and β3 integrins and ECM proteins including the native 70 kDa (rather than the 45 kDa fragment of SPP1), fibronectin, vitronectin and several other members of the small integrin-binding ligand N-linked glycoprotein (SIBLING) family beginning around day 40 of pregnancy and increasing through day 120 (Burghardt *et al*., 2009). Stromal cells in this same tissue compartment of the gravid horn also exhibited upregulation of smooth muscle actin, desmin and vimentin indicative of myofibroblast differentiation. These stromal/myofibroblasts are surrounded by a connective tissue matrix that is more strain shielded due to crosslinking of ECM in three dimensions (3D) compared to the complex forces focused at the maternal conceptus interface (Burghardt *et al*., 2009). These results suggest that FA assembly at the endometial- placental interface and within placentomes and stromal compartments reflects dynamic adaptation to increasing forces caused by the growing conceptus. Cooperative binding of multiple integrins to SPP1 deposited at the endometrial-placental interface form a strong adhesive mosaic to maintain a tight connection and increased tensile strength and signaling activity between endometrial and placental surfaces along regions of epitheliochorial placentation in sheep.

## Conclusions

Elongation and implantation in sheep are complex events that require significant energy, the substrates for which are primarily supplied as histotroph from the uterus. Embryonic mortality during this complex, energy consumptive, peri-implantation period of pregnancy remains a major constraint to improving reproductive efficiency and profitability in livestock enterprises. Sheep are a strong, if niche, livestock industry within the U.S., and consumer demand for lamb meat and wool products is strong. However, the U.S. sheep industry supplies less than half of this demand because reproductive inefficiency in ewes hampers the ability of the U.S. sheep industry to generate quality meat and wool at a viable profit margin (Shiflett *et al*., 2007). Further, sheep are a compelling animal model for the study of cow placental biology. Sheep are less expensive and more easily manipulated experimentally than cattle, while the formation of syncytia in ovine placentomes is thought to be very similar to the initial formation of the syncytial trophoblasts of cow placentae. In cattle, the fertilization rate is 90%, yet the calving rate to a single fertilization is only 55%. This constraint on cattle production is a considerable burden to the U.S. cattle industry. Our understanding of the complex molecular events that underlie successful implantation and placentation across species has been and will likely continue to be advanced by studies of sheep as biomedical research models and to increase reproductive success in animal agriculture enterprises providing high quality protein for humans.
